# Easier comparison of bets in evaluation does not reduce classical preference reversals: Evidence against a context-dependent explanation

**DOI:** 10.1371/journal.pone.0292011

**Published:** 2024-01-03

**Authors:** Raúl López-Pérez, Eli Spiegelman

**Affiliations:** 1 Institute of Public Goods and Policies, Spanish National Research Council, Madrid, Spain; 2 Economics & Social Sciences, Burgundy School of Business, Dijon, France; University of Hertfordshire, UNITED KINGDOM

## Abstract

In preference reversals, subjects express different rankings over a set of alternatives depending on how preferences are elicited. In classical reversal tasks, for instance, subjects often select a safe bet over a risky one when given a choice between the two in a pair, but then assign a higher monetary evaluation to the risky bet. Motivated by a rich literature on context-dependent preferences, we conjecture that comparisons across bets in a pair can influence *both* Choice and Evaluation. Yet deciders are less likely to mentally compare the bets in the latter case, as bets are typically evaluated in isolation. This asymmetry between Choice and Evaluation is, we surmise, one cause of the reversals. If we further assume that memory decay affects mental comparisons in Evaluation, the account predicts order and timing effects on the reversal probability. We run several treatments designed to facilitate or hinder the retrieval from memory of the alternative bet during evaluation of a bet. However, the reversal rate does not vary across treatments in the predicted direction, and we find no systematic order or timing effects. We conclude that reversals are not influenced by the ease with which subjects recall the alternative bet during the evaluations, which suggests in turn that a relatively smaller frequency of comparisons across bets during the (typically isolated) evaluations is not a significant cause of reversals.

## 1. Introduction

Preference reversals (PRs) have represented an empirical puzzle for more than half a century [1–6]. In classical PR experiments, subjects are faced with pairs of binary bets, a $-bet with a larger prize and a P-bet with a larger chance of winning. Their preferences are elicited through two different tasks: *Choice*, in which they indicate which bet they prefer to play, and *Evaluation*, which elicits the certainty equivalent or money value for each bet. The robust empirical pattern is the so-called “standard” reversal: a substantial proportion of participants (often the mode, sometimes the majority) choose the P-bet but assign it a lower value. The causes of such inconsistencies are of general theoretical interest and may also have practical implications. For a first example, consider an agent who allocates resources taking the stated preferences of others into account. Depending on the circumstances, such data may be elicited through surveys, online ratings of restaurants or hotels, marketing focus groups, or even elections. If different methods lead to different stated preference orderings, an understanding of the causal mechanisms driving these differences becomes useful for designing elicitation techniques that reveal the ordering most relevant for the agent’s allocation problem ‒in turn, a better comprehension of the causes can be also useful in discriminating which ordering is most relevant for the problem at hand; e.g., whether some procedures reveal ordering closer to the ‘true’ preferences (assuming such preferences exist at all). For a second example, perhaps closer to our specific research questions, consumers might evaluate a certain good, for instance, a car, differently if it is presented in an isolated manner than if it is compared *in situ* with other cars (e.g., [7]). For regulation authorities this can be important in deciding what information prospective consumers should have.

We use experiments to explore a potential mechanism underlying the standard reversals, based on three intuitions. When choosing between two bets, *first*, people compare those bets, and these comparisons affect the subjective value of each bet and hence Choice. For instance, people may compare payoffs and probabilities across bets, and also attempt to achieve some consistency in the arguments used to infer how desirable each bet is–e.g., if an agent computes the expected value of one bet, he may also decide to compute it for the other available bet. If comparisons are important for Choice, *second*, it is natural to assume that they also influence Evaluation. The problem, however, is that evaluations are performed in isolation in the standard PR designs. In these settings, therefore, achieving the equivalent comparisons in Evaluation as in Choice would require deciders to retrieve or recall the alternative bet. Taking into account that decisions are made in blocks by task in the standard PR designs, such comparisons seem extremely difficult, as they require experimental participants to mentally “pair” bets from a potentially randomized sequence. This is, we surmise, a fundamental asymmetry between Choice and Evaluation, and one potential cause of the PRs, as we explain in more detail below.

Specifically, our framework makes two key assumptions, (a) and (b). (a): Choices and evaluations are reference-dependent. This implies that (at least) some people give different evaluations of the same bet if the alternative bet in the pair, i.e., its reference point, is simultaneously considered than they otherwise would. (b): Consideration of the alternative bet in Evaluation depends on memory and present perceptions, i.e., the context. Consideration is trivial if the alternative bet is part of the context, e.g., displayed together with the bet under evaluation; this is however very rare in the PR literature, as we noted above. Otherwise, consideration requires that the bet is retrieved from memory. Yet such retrieval is subject to decay, and thus unstable.

Assumption (a) is in line with a rich literature on context-dependent preferences, according to which choices and evaluations are shaped by comparisons of the attributes (e.g., the prizes and winning probabilities of two bets) of the options that decision makers have *in mind* [8–12]. One implication of the model by [9], is that reversals should only occur when evaluations take place in isolation. Assumption (a) is also closely related to the Evaluability hypothesis of [13], which holds that human decision processes underweight attributes that are difficult to evaluate in the absence of adequate reference points, potentially leading to reversals (see also [7,14]). For instance, the probability of winning the money prize in a bet is a rather arcane attribute to most agents and hence potentially under-weighted relative to the prize when a bet is evaluated in isolation. Since the P-bet dominates the $-bet on the “probability dimension”, while the $-bet is better on the “prize dimension”, evaluations tend to relatively favor the $-bets, while choices favor the P-bets, which corresponds to the precise form of the standard reversal. Assumption (b), in turn, is a trivial implication of the well-known phenomenon of *decay* in the literature on memory [15–20]. In our context, a subject facing some bet is more likely to compare it with the alternative, reference bet if the latter was *recently* considered or rehearsed–note: we make no assumption on whether agents “attempt” to retrieve the reference bet during the evaluations or just recall it in an automatic, non-volitional manner; both cases are possible a priori.

The mechanism proposed for the PRs directly suggests several research questions, which we explore here. To start, does the availability of information about the alternative bet affect the PR rate? Second, is that rate conditional on the timing and order of presentation of the bets? The theory predicts an affirmative answer to both questions, as these factors influence the likelihood that the relevant reference point is considered in Evaluation. For instance, when pricing, say, the P-bet in a pair, we propose that a decider will be more likely to compare it with the $-bet if she just completed another task involving that $-bet (Choice or Evaluation), or if she has any record of these tasks. By the same token, a delay or distraction between tasks should further cloud reference points and increase the PR rate.

These research questions have not received much systematic attention, although three recent experimental papers address research questions closely related to ours. [21] test the hypothesis, motivated by the Salience Theory of [10], that the evaluation of lotteries in isolation leads to overpricing, particularly of the $-bets. In their experiments, the evaluation of a target lottery L_T_ is elicited in isolation or in the presence of an alternative lottery (a slight variation of the alternative to L_T_ in its pair). Using eye-tracking techniques, they show that attention paid to the alternative lottery reduces the evaluation of L_T_ when it is a $-bet as predicted, but not enough to have a measurable effect on PR rates. The findings, therefore, fail to support Salience Theory. In turn, [22, p.73] contend that “the literature has remained silent on order effects” and hypothesize, based on some psychological evidence, that choices might affect posterior evaluations. In their design, there are 20 pairs of lotteries, i.e., 40 bets in total, and subjects first complete the evaluations of the 40 bets, then the 20 choices, and finally evaluate the same 40 bets again. They report some order effects, although small. Specifically, the rate of *non-standard* reversals–that is, where participants choose the $-bet, but give it a lower monetary evaluation–is higher when the evaluations are elicited before Choice. In contrast, they find no significant order effect on standard reversals. [23] manipulates the availability of the alternative bet during evaluation, finding that standard PRs are much more frequent than nonstandard PRs when the alternative bet is unknown. Note finally that [10] offer some evidence that reversals prevalently occur when evaluations take place in isolation from online, non-incentivized experiments.

Our paper reports several experimental treatments that explore the effect of order, timing, and information, thus testing the relevance of memory-dependent sources of PRs rather generally. Perhaps surprisingly, these effects are not significant in our design. Subjects in our experiment face three pairs of bets. For each pair, they complete Choice and two randomly ordered Evaluations. In the *baseline* treatment (T1, N = 101), the tasks are performed in immediate succession, while two of them are separated by a distractor task in the *interrupted* treatment (T2, N = 93). Since the distractor arguably induces delay, our principles directly predict a higher rate of PRs in T2. A third, *recorded* treatment (T3, N = 96) is identical to the baseline treatment, except that subjects must keep a written record of the bet(s) available in each pair as they appear, together with their choices and evaluations. Because they have reminders of both bets in a pair, the second evaluation in this treatment should be made with reference points essentially the same as those in Choice. We therefore expect the lowest reversal rate in T3. If PRs are due to changes in the “working memory information”, that is, we foresaw a ranking T2 > T1 > T3 in the percentage of reversals. In contrast, we observe a (statistically significant) *increase* in PRs from T1 to T3, and no significant difference between T1 and T2. Further, the frequency of standard reversals in T3 is in fact non-negligible (19%). The order of tasks is balanced across our experimental sessions, but we find that this has little systematic impact on the standard PR rate–as in [22], though, the rate of non-standard PRs rises significantly (from 5.8% to 9.6%, p < 0.05) when the evaluations are elicited before Choice. Overall, these mostly negative results cast doubt on the idea that incoherencies in classical PR experiments are caused by the mechanism proposed. From our point of view, in other words, the reason why PRs occur *in these classical experiments* is not that subjects fail to make comparisons across the bets in a pair during the evaluations; we expand on this point in Section 4. In this respect, therefore, we report confirmatory evidence in line with the study by [21], which found little evidence that the presence of an alternative bet shapes the PR rate.

In the rest of the paper, our contributions are presented as follows. Section 2 describes our experimental treatments and develops our research hypotheses. Section 3 analyzes the data in the light of our hypotheses. Section 4, in turn, discusses some potential objections to our design. We conclude, as said, with a discussion of our results but also of some potential future research.

## 2. Experimental methods and research hypotheses

During the computerized experiment, subjects face a total of six bets, grouped in three pairs (see Table 1). In each pair there is a P-bet and a $-bet, as defined above, although these terms were not used in the instructions. Probabilities are expressed as multiples of 1/30 (for example, 10/30 to win €24.00), and interpreted with reference to a bag with 30 numbered balls. In each pair, the bets have a similar expected value. Still, following [24], the expected value of each P-bet is slightly lower than that of the corresponding $-bet in the pair. In this manner, we do not favor the choice of the P-bets and hence increase behavioral variability.

**Table 1:** P and $-bets employed in the experimental design.

Pair	P-bet	Expected value	$-bet	Expected value
1	(29/30, 8)	7.73	(10/30, 24)	8
2	(22/30, 10)	7.33	(8/30, 30)	8
3	(26/30, 9)	7.8	(3/30, 80)	8

**Note**: The pair (p, x) denotes a bet with a probability p to earn x Euros, and nothing otherwise. Each expected value refers to the bet immediately on the left.

The specific bets chosen were adapted from Set I in [24], with prize values roughly doubled and probabilities expressed as multiples of 1/30 rather than 1/36 as in that paper. Our Pair 3, for instance, is nearly identical to their Pair 4 with doubled prize values, and pairs 1 and 2 in our study fall within the range of a re-scaling of the other bets from Set I. We note that [23] also uses a re-scaling of these same bets in his study.

For each pair of bets, subjects perform the same three *primary* tasks. These include two Evaluation tasks, which elicit a cash equivalent of each bet in the pair, and a Choice task where they are asked to choose between the two bets or to express indifference between them. For incentive compatibility, the subjects are told that, at the end of the experiment, the computer will randomly select (a) one of the three pairs of bets and (b) the type of task (i.e., either Choice or Evaluation). In that (a) pair and (b) task, subjects play the bet that they previously favored, i.e., the bet they preferred in Choice or the one assigned a higher cash equivalent in Evaluation; if they had expressed indifference in the selected pair and task, one of the bets in that pair would be drawn at random to play. Thus, we use the ordinal pricing mechanism (OPM) as in [24]–see also [25]. Paying for just one bet rules out potential portfolio effects in the choice of lotteries.

Tasks were organized largely in pair-wise triples, rather than in blocks of each task as is common in the literature. Subjects therefore completed all the tasks for one pair of bets, then received an indication that the following tasks related to another pair, before moving on. See Section 4 for a detailed argument of why we chose this method and our specific pairs of bets.

We make one additional point in passing about the OPM. If a subject is indifferent between the two bets P and $ in a pair, he might theoretically enter any two money evaluations E_P_, E_$_ for the respective bets. In effect, whatever the ranking of the stated evaluations (i.e., E_P_ > E_$_, E_P_ < E_$_, or E_P_ = E_$_), the subject will always play her favorite bet if Evaluation is chosen for payment, simply because both bets are equally good, given indifference. It is for this reason that it was key for us to introduce the possibility of indicating indifference in Choice. Otherwise, participants might enter apparently conflicting answers without true violation in cases of true indifference. However, it must be noted that allowing this explicit indifference is not common in the previous literature. Further, we will define reversals as strict inconsistencies, so allowing indifference gives participants a way out of reversing, which might reduce the measured rates of PRs in our results compared to previous studies. In any case, however, this seems to be immaterial for the test of our empirical hypotheses below.

Our experiments seek to manipulate the information about the alternative bet that participants have in mind during the evaluation tasks. Common to all treatments, the sequence of what we call the three *primary tasks* (Choice and Evaluation of each bet) vary between bet pairs for each participant. In addition, we implement four different treatments to facilitate or impede the maintenance of the alternative, reference bet in mind. Specifically, Treatment T1 (*baseline*) has participants complete the three primary tasks for each pair in immediate succession. By contrast, treatment T2 (*interrupted*) separates the first and second primary tasks (which, again, varied from pair to pair and subject to subject) with an unrelated distractor task. In this task, subjects are shown an image with four randomly generated dots on a 3x3 grid corresponding to the numbers on their computer keyboard (for further detail, consult the online appendix). They must identify and add the numbers together, entering the sum on the computer. This requires them to map the keypad to the image on the screen and perform a simple mental calculation. They are paid €0.50 for every second sum correctly entered and must answer the calculation correctly before they can move on to the next. For instance, completing either four or five sums earns a participant €1, while completing six earns €1.50. In each distractor task, they have 40 seconds to do as many sums as they can. To control for fatigue and potential wealth effects, participants in T1 and T3 also complete the same distractor task before the first of the three primary tasks of each pair. Since the distractor task demands attention and effort, and subjects have an incentive to exert that effort, in T2 we expect them to divert attention from the first primary task while they complete the sums. In other words, this diversion acts not only as a filler to induce delay: by taxing working memory and blocking rehearsal, we conjecture it to hinder recall of the details of the first primary task. The empirical hypothesis thus predicts a lower rate of PRs in T1 than in T2:

**EH1:** The PR rate increases if subjects complete the primary tasks at longer intervals (provided that they do not consider or rehearse details of the prior tasks in the meanwhile).

Treatment T3, or *recorded*, was identical to T1, except that it required participants to keep a written record of their decisions as they went. Specifically, we asked subjects to draw the available bet(s) in each primary task as well as indicate their corresponding choice/evaluation (see the instructions in the online appendix for further detail). Table 2 below illustrates the differences between treatments in the flow and type of tasks for a given pair of bets.

**Table 2:** Order and type of tasks in each treatment, for a given pair of bets.

Stage	Treatment		
	T1	T2	T3
**1**	Distractor task	First primary task	Distractor task
**2**	First primary task	Distractor task	First primary task & written record of choices
**3**	Second primary task	Second primary task	Second primary task & written record of choices
**4**	Third primary task	Third primary task	Third primary task & written record of choices

**Note**: A primary task is either the Choice between the two bets in the pair or the Evaluation of one of the bets. The order of tasks was balanced across pairs.

The experimenter in T3 could easily monitor whether subjects completed the drawings, as the sessions were run in a small room with 12–14 subjects per session. In addition, subjects had to hand over the sheets with the drawings after the experiment finished, which allowed us to check that subjects had acted as required in the instructions. The goal of T3 was to increase awareness or facilitate retrieval of the alternative bet during the evaluations in a pair (except in those evaluations that were the first primary task in a pair, in which case there were no reminders). T3, in contrast to T1, did not just leave recall unimpeded, but in fact favored it. T3 allows us to test

**EH2:** Subjects who complete one task while being shown the results of previous ones will have fewer reversals than those who complete the tasks in isolation.

In other words, EH2 predicts a lower rate of PRs in T3 than in T1. For a motivation of our last treatment, one might object that even if subjects in T2 (Interrupted) were not thinking about the details of their first task during the distractor task, the subsequent primary tasks may have recalled them to mind by association. For example, suppose that the decision maker in the Interrupted treatment first chooses between the P- and $-bets, and then evaluates one of those bets after the distraction. This bet could act as a cue, recalling the memory of the choice task and hence the alternative bet, and thereby refreshing the alternative as a reference point. Something similar would be expected in the second evaluation. Since the referents would be the same across tasks, the likelihood of a PR in this specific example should be unvaried in T1 to T3. In a nutshell, the argument says that decay is irrelevant if the order of the tasks allows for associative recall. While our priors were hardly that strong, we have two remarks in this respect. To start, the argument still predicts a clear order effect. If the first task in a pair is Evaluation, then obviously that association could not bring the other, as-yet unseen bet to mind, so that the referents would vary across tasks. This point will be checked in detail later (see hypothesis EH3 below).

Second, as an additional robustness check, T4 repeated T1, but with a fully randomized sequence of the nine primary tasks (3 tasks x 3 pairs). That is, in T4 (*random*) subjects completed the Evaluation for each of the six bets, and Choice for each of the pairs from Table 1, but it was pure coincidence if any three sequential tasks related to the same pair of bets: tasks were not “blocked” at all, either by task or by pair. As in T1, further, subjects completed a distractor task before the first, fourth, and seventh primary tasks. From our point of view, association-based recall based on temporal contiguity [19,26] should be weak in T4, as the bets in a pair are often presented at a distance. Since neither decay nor associative memory help to keep the referents constant across tasks in a pair, one should expect a higher frequency of PRs in T4 than in the other treatments. Note finally that T4 allows for further tests of Hypothesis EH1 above, as it induces substantial variation in the delay between tasks within pairs of bets.

Order effects were investigated through systematic manipulation of the sequence of tasks within any bet pair (see Table 3 where Choice = C and Evaluation = E). There are six possible *histories* for any bet pair, defined by (1) the order of the two Evaluation tasks, and (2) the placement of choice among them. The Evaluation order was randomized at the individual level for each bet pair; Choice placement is balanced across bet pairs through three *sequences* implemented at the session level. For a given pair (sequence) Choice is the first, second or third task, depending on the sequence (pair). Note that each cell in Table 3 will induce two different histories, depending on the individual-level randomization of the order of the P-bet and $-bet. For instance, in Sequence S1, Pair 1 always has Choice followed by two evaluations, but this leads to the specific histories (C-$-P) and (C-P-$). These same histories are induced for Pair 2 in Sequence 3, and for Pair 3 in Sequence 2. Sequences were balanced at the session level across treatments T1-T3, so treatment effects should be independent of session effects.

**Table 3:** Ordering of the primary tasks in each bet pair; different sequences.

		Bet Pair			N
		1	2	3	
**Sequence**	**S1**	C–E1 –E2	E1 –C–E2	E1 –E2 –C	99
	**S2**	E1 –C–E2	E1 –E2 –C	C–E1 –E2	97
	**S3**	E1 –E2 –C	C–E1 –E2	E1 –C–E2	94

Note: C refers to the Choice task, and E1 and E2 to the Evaluation of the two bets, whose order was randomized at the individual level in each sequence. N refers to the number of subjects observing each sequence.

Note that our basic hypothesis is that easier retrieval of the alternative bet affects Evaluation of bets carried out in isolation. If participants had all the information about the bet pairs in mind (as they presumably do in Choice), then they would (presumably) reveal the same preference ordering in both tasks. In this sense, reversals are “errors”: participants would not commit them if they had consistent reference points across tasks. In Choice, since both bets in the pair are simultaneously visible, this mechanism cannot lead to such errors. This argument therefore defines the “correct” ordering as that revealed in Choice, which is equivalent to assuming that Choice is made without “error”. It follows that predictions from our order effects are relevant only to the Evaluation decisions. This means that although our central interest for the analysis is reversals, the effect should come through changes in the evaluation of the bets. We can therefore investigate these directly, which leads to our third empirical hypothesis.

**EH3:** History of tasks at the time of evaluation will affect the value attributed to each bet.

We ran 24 computerized sessions (seven each of T1, T2, T3 and three of T4), with 12 run at Universidad Autónoma de Madrid (*N* = 149) in December 2014, and the remainder at the Université de Montpellier (*N* = 176) in June 2021, for a total of 325 participants. The treatment sample sizes were 101, 93, 96 and 35 for treatments T1-T4, respectively. All participants were recruited voluntarily through standard procedures, and the project was approved by Ethical Review Boards at both authors’ institutions. Written informed consent was secured through a page of the online registration platform for the participants in Montpellier, and a signed consent form in Madrid. The software used was z-Tree [27]. Participants were not students of the experimenters, and each participated in just one treatment. After being seated at a visually isolated computer terminal, each participant received written instructions that described the decision problem (see the online appendix). Subjects could read the instructions at their own pace, and we answered their questions in private. The instructions were then read aloud and understanding of the rules and particularly of the payment procedure was checked with a control questionnaire that all subjects had to answer correctly before they could start making choices (consult the online appendix).

After all tasks had been performed by each subject, payment was determined according to the protocol described, and the subjects informed of any winnings. Afterwards, they answered a brief questionnaire where we gathered information on socio-demographic characteristics, as well as the cognitive reflection test or CRT [28]. This ended the experiment. Subjects were paid in private. Each session lasted approximately 60 minutes, and on average subjects earned 12.71 Euros, including a show-up fee of 4 Euros.

Finally, note that consistency in the primary tasks may be influenced by other factors than basic memory capacity. For instance, being alert and mentally active will presumably aid the process, as will underlying numeracy skills. Relative to the first point, the results of the CRT appear to be a good measure of how willing someone is to meditate on a problem [28]. The second will likely be reflected in relative performance in the distractor task, which required rapid mental arithmetic. We can therefore test both of these associations directly, using, respectively, the score on the CRT test and the number of sums correctly completed.

**EH4**: Reversals will be more likely in subjects who perform relatively badly in the distractor tasks and the CRT measures.

## 3. Data analysis

Since each experimental participant considered three pairs, there are two different units of observation that can be used: the bet Pair level and the participant level. At the Pair level, there are two main attributes of interest with regards to the PR phenomenon: which bet was picked in Choice, and which one was given the higher evaluation. A *strict standard reversal* occurs when the P-bet is indicated as strictly preferred and given a strictly lower evaluation. A *weak standard reversal* occurs when either the P-bet is at least as good as the $-bet in Choice and gets a strictly lower evaluation or gets an evaluation not higher than that of the $-bet but is strictly preferred. *Strict (weak) non-standard reversals* are defined analogously for participants who expressed strictly (weakly) preferring the $-bet in Choice yet gave it a strictly (weakly) lower rating in Evaluation (with at least one choice being strict). In this paper we will generally focus on strict standard reversals, and unless otherwise specified, the terms *reversal* and *PR* will refer to those, although none of our results are qualitatively different for weak standard PRs. The patterns of non-standard reversals are somewhat different; they are not our main focus but will be identified when they are of interest.

Because each participant completed three bet pairs, not all bet-pair-level observations can be treated as independent. Our regressions use random-effects panel models, clustering standard errors at the individual level to correct for this, but more directly, we investigate outcomes at the participant level, where all observations should be independent. In this case, our main outcome of interest will generally be the number of reversals committed, a number between zero and three.

Bet pair-level outcomes are summarized in Table 4, allowing a first look at how the aggregate choices corresponded with the standard reversal phenomenon. The bottom row shows that overall, the P-bet was chosen over the $-bet in just over 60% of pairs (610/975), and the $-bet was given a higher rating in just over 40% (398/975). Nearly thirty percent of all pairs of bets resulted in at least a weak standard PR, and over one in six resulted in a strict standard reversal. The rate of strict non-standard PRs was 0.07 (weak 0.12). Interestingly, however, conditional on Choice behavior, the rates were more similar. For instance, restricting attention to the 908 bet pairs where one bet was chosen as strictly preferred, 65.4% (195/298) of those having chosen the $-bet also rated the $-bet strictly higher, while 58.5% (357/610) of those having chosen the P-bet also gave the P-bet a strictly higher rating. A Pearson Chi-square test (df = 1; p = 0.045) still shows that choosing the P-bet is associated with less consistent behavior–that is, standard PRs are more common than non-standard–but the difference is much smaller.

**Table 4:** Rates of behavioral patterns across treatments.

Treatment	N (indep)	Choices			Evaluations			Standard PR rates		Non-standard PR rates	
		**$-bet**	**Ind.**	**P-bet**	**V**_**$**_ **> V**_**P**_	**V**_**$**_ **= V**_**P**_	**V**_**P**_ **> V**_**$**_	**Strict**	**Weak**	**Strict**	**Weak**
**Baseline (T1)**	**303 (101)**	0.277	0.069	0.654	0.347	0.139	0.515	0.139	0.271	0.079	0.122
**Interrupted (T2)**	**279 (93)**	0.330	0.061	0.609	0.416	0.147	0.437	0.161	0.305	0.075	0.133
**Recorded (T3)**	**288 (96)**	0.319	0.080	0.601	0.472	0.139	0.389	0.194	0.319	0.049	0.101
**Random (T4)**	**105 (35)**	0.286	0.057	0.657	0.391	0.114	0.495	0.229	0.286	0.086	0.162
**Total**	**975** **(325)**	**0.306**	**0.069**	**0.626**	**0.408**	**0.139**	**0.453**	**0.171**	**0.296**	**0.070**	**0.123**

Table 5 tells a similar story at the individual level. In every treatment there were both individuals who managed not to commit any PRs at all (indeed, these were always in the majority), and a few who reversed on all three pairs. In fact, the 14 individuals –4.3% of the overall sample–who committed three PRs each accounted for one quarter of all PRs observed in the experiment.

**Table 5:** Frequency of strict standard PRs across treatments.

# of Reversals	Treatment				Total
	T1 –Baseline	T2—Interrupted	T3 –Recorded	T4—Random	
**0**	76	64	57	20	217
	(75.25)	(68.82)	(59.38)	(57.14)	(66.77)
**1**	13	17	24	9	63
	(12.87)	(18.28)	(25)	(25.71)	(19.38)
**2**	7	8	13	3	31
	(6.93)	(8.6)	(13.54)	(8.57)	(9.54)
**3**	5	4	2	3	14
	(4.95)	(4.3)	(2.08)	(8.57)	(4.31)
**Total**	101	93	96	35	325
	(100)	(100)	(100)	(100)	(100)

We use these data to investigate our first two empirical hypotheses. We recall the first below:

*EH1*: *The PR rate increases if subjects complete the primary tasks at longer intervals (provided that they do not consider or rehearse details of the prior tasks in the meanwhile)*.

Inspection of Tables 4 and 5 indicates that, consistent with EH1, the Interrupted and Random conditions have increased reversal rates over the Baseline. In Table 5, for instance, the proportion of “perfect score” participants is larger in T1 than in either T2 or T4: participants do better when they complete all tasks for a bet pair in immediate succession. However, the differences are not statistically significant. At the individual level, a Pearson Chi-square test of the association between the number of strict reversals and treatments fails to detect a significant relationship either between T1 and T2 (p > 0.4), or T1 and T4 (p > 0.2). At the level of the bet pair, Table 6 reports coefficient estimates for random-effects logistic models predicting strict reversals as function of treatment comparisons. They show that neither T2 (regression 1) nor T4 (regression 2) induced significantly more probability of a PR than did T1. We also report the estimated effect of performance in the CRT and the distractor task, which are related to hypothesis EH4, discussed below. Standard errors are clustered at the individual level, and there are unreported controls for gender, age, the sessions run in France, and the individual bet pairs. There were somewhat fewer reversals in the second wave of sessions, and in Pair 3. Controls for gender, age CRT and performance in the distractor task were insignificant.

**Table 6:** Random-effects panel (logistic) regressions of reversal probability on treatment variables.

	(1)	(2)	(3)	(4)
Test	EH1	EH1	EH1 –T4	EH2
	T1 v T2	T1 v T4	VarPair	T1 v T3
**Treatment effect**	0.468	-0.0590	-0.0707	0.391**
	(0.499)	(0.242)	(0.0722)	(0.196)
**CRT (no. correct)**	0.0138	0.00783	0.0775	-0.0197
	(0.0528)	(0.0577)	(0.0917)	(0.277)
**Score in distractor task**	-0.170	-0.296	0.0702	-0.0630
	(0.365)	(0.457)	(0.780)	(0.0413)
**Constant**	-1.140	0.532	2.610	-0.256
	(1.572)	(2.444)	(4.828)	(1.142)
**Observations**	582	408	105	591
**Number of PID**	194	136	35	197

**Note**: Coefficient estimates for random-effects logistic regressions, with strict reversals as the outcome variable. Treatment effect refers to the difference between indicated conditions except in regression (3), where it is the temporal spread between tasks associated with the pair, i.e., the *Varpair* variable. Robust standard errors in parentheses. Controls include gender, age, a dummy for the French round of sessions, and dummies for the different pairs of bets.

** p < 0.01

* p < 0.05.

Regression (3) exploits the fact that in T4 the order of the nine tasks (three Choice tasks and six Evaluation tasks) was completely randomized, separately for each individual, which means that the spread, or temporal displacement of each bet pair, is also variable, and moreover orthogonal to individual characteristics across pairs. To begin, we assign each task a number reflecting its order among the nine. We then calculate the variable *VarPair*, which is simply the variance of the task numbers associated with a particular pair. For an example, suppose that the three tasks in Pair 1 come in positions 1, 7, and 9 (out of 9 positions). Then VarPair for this Pair 1 equals V({1,7,9}) = 17.3. Since the task numbers are unique integers between 1 and 9, *VarPair* takes values between 1 (if all tasks for a pair are completed sequentially) and 19 (if the first, last and second or second-last tasks are all for the same pair); the observations we have cover this range entirely. (Choice times would have given finer-grained information on this delay; our data did not include this information, and at any rate the correlation with the task order would be very high.) Since EH1 states that temporal splits between tasks are likely to generate PRs, it predicts a higher likelihood of reversals for those pairs with a higher spread of the tasks. Yet the results of the regression find no such effect. Our conclusion is that **within the range of time implemented in this experiment, delay between the tasks and/or the blocking of rehearsal caused by the distraction task does not predict reversals.** We now turn to the next hypothesis.

*EH2*: *Subjects who see previous results while completing each task will have fewer reversals than those who complete the tasks in isolation*.

Our treatment T3, which required participants to maintain a record of their decisions, explores this possibility. Comparing PR rates in the T3 (recorded) and T1 (baseline) conditions in Table 4, we see that, contrary to our hypothesis, in fact reversals increase slightly in T3 relative to the baseline. Indeed, regression (4) of Table 6 indicates that the difference is statistically measurable. This relationship holds at the individual level as well: Table 5 reveals that substantially more participants in T1 committed zero reversals than in T3 (75% versus about 60%), and nearly twice as many committed one or two reversals in T3 as in T1 (38% as opposed to 20% in T1). A Fisher’s exact test of equality of distribution of PRs across the T1 and T3 conditions confirms that there is a significant relationship between the variables (p < 0.05). Whatever the reasons for this intriguing result, the conclusion is that **a record of previous decisions does not reduce reversals.** Thus, EH2 is also not confirmed.

In turn, EH3 concerned the effect of different task histories on the evaluation of the bets. We recall it below:

*EH3*: *History of tasks at the time of evaluation will affect the value attributed to each*.

Because (1) the sequence of bets was balanced across treatments T1 to T3; (2) the mechanism of EH3 should work in all treatments; and (3) at any rate, behavioral differences were limited, we pool these three treatments together to investigate EH3. Table 7 shows the average evaluation of the $- and P-bets across all bet pairs in conditions T1-T3, and the rate at which the P-bet was preferred in Choice, depending on the specific history observed.

**Table 7:** Behavioral effects of task sequence.

Order	N	Sequence of tasks	Evaluation of $-bet	Evaluation of P-bet	Chose P-bet	$-bet preceded by	P-bet preceded by
1	149	C-$-P	11.36	13.80	0.73	Choice	Both
2	141	C-P-$	10.52	13.12	0.81	Both	Choice
3	153	$-P-C	11.14	11.94	0.73	Nothing	$-bet
4	143	$-C-P	11.25	11.48	0.81	Nothing	Both
5	137	P-$-C	11.37	14.33	0.76	P-bet	Nothing
6	147	P-C-$	15.98	13.06	0.73	Both	Nothing
Any	870		11.95	12.94	0.76	----	----

**Note**: Order shows the sequence of tasks, with $ (P) indicating evaluation of the $-bet (P-bet), and C indicating the Choice task. Cells indicate the average evaluation across all three bets, and proportion choosing the P-bet in Choice.

One remarkable point is that the value of the $-bet evaluation in order 6 (i.e., P-C-$) is substantially higher than in any of the other orders. Although a Mann-Whitney test of these evaluations against those pooled from other histories does not indicate significance (p > 0.05), this might suggest that evaluating the $-bet last increases the value attached. Yet notice that order 2, (C-P-$), which also puts evaluation of the $-bet last, but inverts the order of the other two tasks, resulted in the *lowest* average $-bet evaluation. The order-2 and order-6 evaluations of the $-bet are moreover significantly different (t(246) = -2.47, p < 0.05). In turn, this raises the possibility that evaluation of the $-bet is not affected by placement, but by juxtaposition with choice or evaluation of the P-bet. On the other hand, compare histories (C-$-P) and (P-$-C), which also have the $-bet evaluation immediately preceded by the Choice task and P-bet evaluations, respectively: in these cases, the evaluation of the $-bet was identical. If there is a stable mechanism generating these results, it may be more subtle than our design allows us to identify. We leave this to future research to investigate.

**Fig 1:**
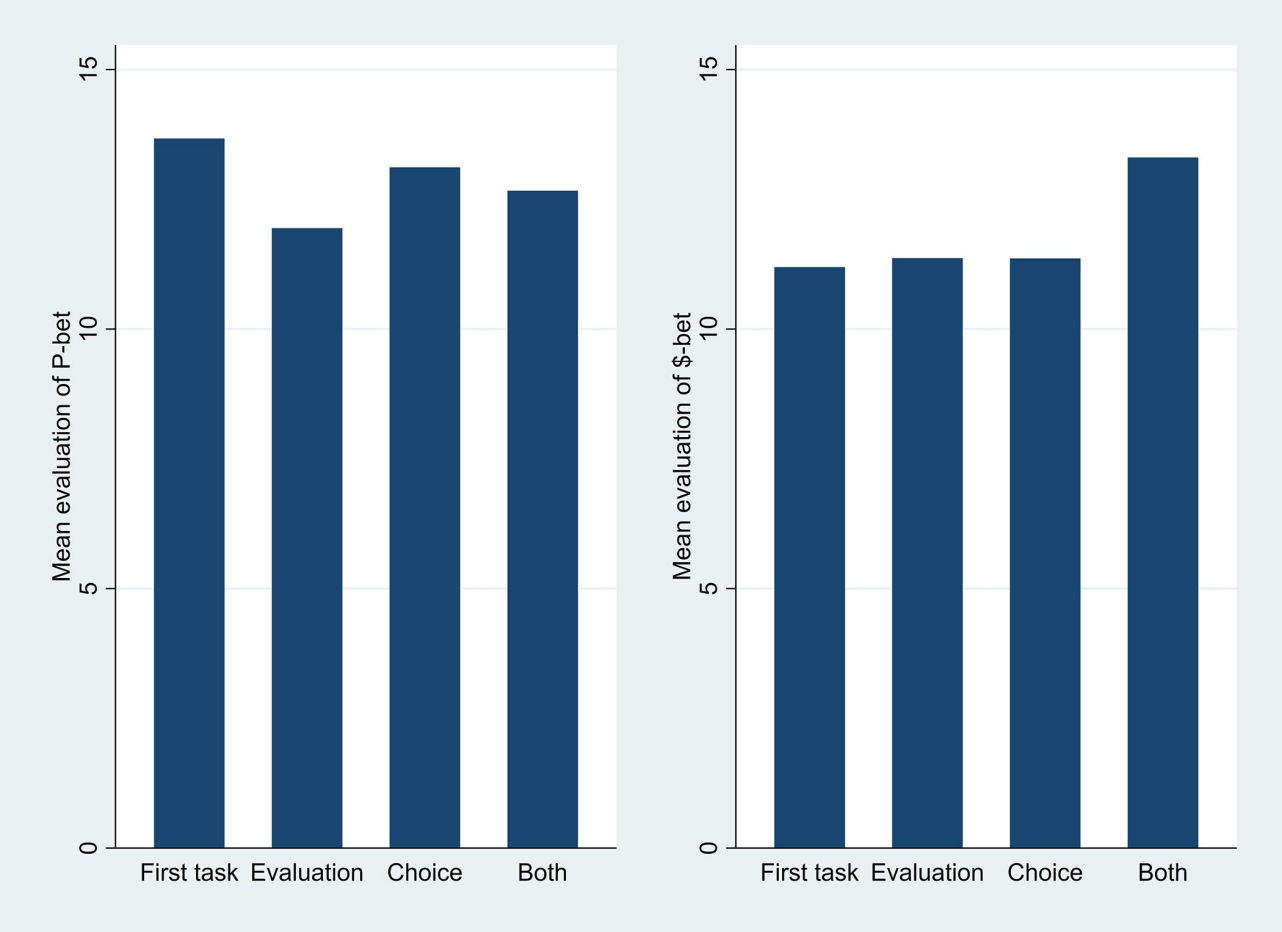
Average evaluation of the bets, depending on prior task within the pair of bets. (a) P-bet preceded by (b) $-bet preceded by.

On a more aggregate analysis, our central statistical test reduces the dimensionality of the histories to four. Upon performing any evaluation, specifically, we note that participants may have just experienced (a) nothing (if that evaluation is the first task); (b) evaluation only of the other bet; (c) only the Choice task; or (d) both of the other tasks. These histories are indicated in the final two columns of Table 7. Because the sequences were balanced across pairs, each order is a mix of pairs; overall there is no relationship between the order of the tasks and the specific pair (Pearson Chi-square (10) = 10.257, p > 0.41). Fig 1 shows the average evaluations for the P-bet (left) and $-bet (right) depending on the histories as defined. We see that history made little difference to the evaluations.

In turn, Table 8 reports random-effects panel regressions of the evaluation of each bet (models 1 and 4), and the probability of a strict standard reversal (models 2 and 5) or a non-standard reversal (models 3 and 6), on indicators for the different histories. We again consider four different histories; each regression therefore has three different history indicator variables; the no-history case (i.e., the evaluation is the first task in the pair) is the baseline. Specifically, models (1–3) use the histories starting with the P-bet evaluation as the baseline, while (4–6) compare to the histories starting with the $-bet. The comparison histories are indicated in the rows. The central message of the table is that none of these histories result in significantly different behavioral outcomes–whether it be evaluation of the bet, or probability of standard or non-standard reversals–than their respective baselines. We have also analyzed the effect on PRs and evaluations of the task that immediately precedes the given evaluation, see the online appendix for more information. Results are in general similar. In particular, prior task has no effect on the likelihood of either standard or non-standard reversals.

**Table 8:** Regression results on order effects.

Test Histories	Base = {P-$-C, P-C-$}			Base = {$-P-C, $-C-P}		
	(1)	(2)	(3)	(4)	(5)	(6)
	P-bet Eval.	Standard reversal	Counter reversal	$-bet Eval.	Standard reversal	Counter reversal
{$-P-C}	-0.310	0.0518	0.464			
	(1.113)	(0.386)	(0.402)			
{C-P-$}	-0.472	0.236	-0.177			
	(0.936)	(0.354)	(0.414)			
{C-$-P, $-C-P}	0.253	-0.150	-0.527			
	(1.126)	(0.308)	(0.384)			
{P-$-C}				-2.109	-0.365	0.225
				(1.148)	(0.409)	(0.391)
{C-$-P}				-1.859	-0.166	-0.760
				(1.062)	(0.369)	(0.531)
{C-P-$, P-C-$}				0.671	0.295	-0.376
				(0.890)	(0.307)	(0.367)
Constant	3.926	-0.522	-3.269**	7.160	-0.557	-3.173**
	(5.771)	(1.016)	(0.745)	(4.555)	(1.042)	(0.703)
Observations	870	870	870	870	870	870
Number of PID	290	290	290	290	290	290

**Note**: Coefficient estimates for random effects OLS (models 1 and 4) and logistic regressions (remaining models). Robust standard errors in parentheses. Controls include scores on the CRT and distractor task, as well as age, gender, a dummy for the French sessions, and indicators for the different bets.

** p < 0.01

* p < 0.05.

Individual-level data with respect to EH3 makes use of the fact that each participant was presented with the primary tasks in different orders, and the order of the evaluations was randomized across subjects. Because of this, each participant had one or two of three pairs in which the evaluation of each bet occurred before choice, and the remaining in which it occurred after, and this variation was balanced across the bet pairs. Across all participants, therefore, EH3 implies that the average evaluations done after Choice should be different from those done before. To investigate this, for each subject we first calculate the average evaluation of bets performed before Choice, and the average evaluation after Choice, and test equality of these averages in a matched-pairs design at the individual level. We find differences neither for the $-bet (Wilcoxon signed-rank test p > 0.6) nor for the P-bet (p > 0.1). Our result hence is that **order effects are of minimal importance in our design.**

With respect to EH4 –*reversals will be more likely in subjects who perform relatively badly in the distractor tasks and the CRT–*we note first that there was a significant relationship between the CRT score and the Sums of the distractor task. The Spearman rank correlation coefficient between the number of Sums completed and number of correct questions on the CRT was 0.437, which was highly significant (p < 0.001). As one would expect, given the mathematical nature of the CRT questions, those who are more fluent with the mental arithmetic involved in the distractor tasks also do better in the CRT.

However, with respect to PRs, the regressions in Table 9 show that neither CRT nor sums are predictive either of strong or weak reversals, regardless of whether measured in a panel logistic specification at the Bet Pair level or in a linear model on the number of PRs committed by each individual. Our conclusion is that **neither CRT scores nor performance on the distractor task predicts reversals.** This suggests that PRs in our experiment are not correlated with the individual’s reflective or numerical capacities.

**Table 9:** Relationship between CRT and distractor tasks on PRs.

	Strict reversal	Weak reversal	Number of PR
**CRT score**	-0.00767	-0.0338	-0.0106
	(0.216)	(0.151)	(0.0345)
**Sums**	-0.0121	0.00197	-0.000536
	(0.0331)	(0.0240)	(0.00523)
**Sex**	-0.311	-0.196	-0.0790
	(0.339)	(0.247)	(0.0550)
**Age**	-0.0525	-0.0252	-0.0117**
	(0.0346)	(0.0205)	(0.00361)
**Constant**	-1.039	-0.936	0.884**
	(1.015)	(0.602)	(0.129)
**Observations**	975	975	975
**R-squared**			0.016
**Number of PID**	325	325	

**Note:** Robust standard errors in parentheses.

** p < 0.01

* p < 0.05.

So far the results have been consistently negative. We finish with a potentially interesting observation, although not directly related to our formulated hypotheses. It concerns differences across the three pairs of bets. We make two remarks here. First, while our econometric analysis controlled for differences in level across the three pairs, it also revealed a tendency–not highlighted in the reports above–for fewer PRs to be committed in the third bet pair than in the first. Table 10 presents descriptive data indicating that the rates at which the P-bet was chosen in the Choice task, and given a higher Evaluation, both slightly rose across pairs, which explains the falling PR rates.

**Table 10:** Behavioral differences across bet pairs.

Bet pair		P-bet			$-bet			C = P	E = $	Reversal rate
	N	Win	Prob	V_P_	Win	Prob	V_$_			
**1**	325	8	0.97	13.777	24	0.33	11.324	0.742	0.428	0.197
**2**	325	10	0.73	11.828	30	0.27	11.397	0.751	0.425	0.172
**3**	325	9	0.87	13.095	80	0.10	13.473	0.797	0.372	0.145
**Total**	975	9	0.86	12.900	44.67	0.23	12.064	0.763	0.408	0.171

Note:

V_j_ is the average valuation of bet j in the pair (j = P, $); C = P refers to the percent of subjects choosing the P-bet in the corresponding pair; E = $ to that giving a strictly higher valuation to the $-bet in the pair.

Since these bets were always presented in the same order, any change in reversal rates over time may be due either to inherent differences in the bet pairs themselves, or to learning effects. Which one was the main driving factor? While this question was not central to our design, we can explore it with a comparison of the first three treatments with T4, in which the order was completely randomized. Consider a regression of the PR probability on the bet pair variable (which can also be thought of as a “period”, since pair order was not varied) in the data from T1-T3. Clearly the estimated effect will be a combination of the effect of the bet itself with a learning curve. In T4, the estimate can only include the former. Therefore, the difference between the bet pair effect in T1-T3 and that in T4 can disentangle the effect of time alone. We estimate this with the interacted regression equation

$$Reversal=\beta_{R}T4+\alpha +\sum_{j=2}^{3}\beta_{j}P_{j}+\sum_{j=2}^{3}\beta_{Xj}(P_{j}\times T4)$$ (1)

where *P*_*j*_ is a dummy for bet pair *j*, and T4 is a dummy for that treatment. In this specification, the constant will be the PR rate for Pair 1, and idiosyncratic characteristics of any given bet pair that generate differences in reversals across all treatments will be captured in the β_*j*_ estimations. The coefficient β_*R*_ will capture the purified time effect. We ran this regression in a marginal probit specification, where the interpretation of the coefficients is the effect of a discrete change of the relevant dummy variable. The time effect of T4 was a reduction in reversal probability of 0.0486, (p > 0.3), suggesting that learning is not very relevant. Coefficients on the β_*j*_ confirm that while Pair 2 had a similar reversal propensity to Pair 1 (coeff. = -0.0388, p > 0.1); Pair 3 appears to have been “easier”, in the sense of a lower probability of reversals, i.e., less inconsistencies or “mistakes” (coeff. = -0.0628, p < 0.05).

The finding that there are inherent differences in the “difficulty” of our bet pairs leads to the second question, which is whether our previous aggregate findings regarding timing of tasks (EH1), feedback about prior tasks (EH2), and order of tasks (EH3) also hold true if we focus the analysis at the pair level, for each bet pair separately. An additional motivation for a disaggregated analysis might concern “spillovers” between bets, due to an accumulation of references from exposure to preceding bet pairs. Still, we doubt the latter to be a very significant effect because it would have been uncovered in the time trend of the estimation of Eq (1). By the same token that learning did not seem to affect reversal probabilities, spillovers are also apparently of limited relevance, at least in our context.

We re-ran the analysis for EH1-EH3 on subsamples of the data consisting of each bet pair individually. Unadjusted p-values for our hypotheses on the effect of the T1 versus either T2 or T3 are insignificant; neither EH1 nor EH2 appears to hold at the individual pair level. This again suggests that any disaggregated effects should come through the channel of inherent differences in the bet pairs, rather than accumulation of reference points. When investigating EH3 on different histories at the individual bet pair level, we find some, rather weak, evidence of effects. For instance, strict reversals are less likely for Pairs 2 and 3 (but not Pair 1) when the $-bet is evaluated last than when it is first. That is, there are fewer reversals *in pairs 2 and 3* following the history set {C-P-$, P-C-$} than the history set {$-P-C, $-C-P} (unadjusted p < 0.05). Still, note that the analysis amounts to testing a family of hypotheses from the same data (one for each Bet Pair, plus the overall); a Holm-Bonferroni [29] correction eliminates all the significance found.

## 4. Discussion

Several objections can be made to our design. First, paying just one bet makes the overall decision context a compound lottery: First one of three bets is randomly selected, then one of two choice tasks. Finally, the resulting outcome is of course also a lottery itself. [30] showed that reversals could be explained by violations of the so-called *compound axiom*, which posits the preference-level equivalence of a multi-stage lottery with a single-stage one that has the appropriately compounded values. However, reversals caused by violations of this axiom should not depend on timing, order, or feedback features, so such violations should not interfere with our treatment effects (if any). At any rate, this practice is common to nearly all incentivized PR experiments we know of.

In contrast, our design differs in two related ways from the standard experimental setup in most of the PR literature. First, the tasks were completed pair-by-pair, rather than in blocks of Evaluation and Choice. That is, in the greater part of our analysis (except in T4), subjects will have completed all three tasks for one pair of bets before moving on to the analogous tasks for another pair. This was necessary to generate precise control over timing and order effects at the pair level. In the standard task-wise block design, in contrast, the tasks specific to each pair will be separated by a substantial number of extraneous decisions from the others, except potentially for a special case if the last task in one block corresponded to the bets from the same pair as the first task in the next. Under the task-wise block design, therefore, retrieval of the alternative bet seems very unlikely even in a baseline condition without distractors, and order effects negligible as well. Even keeping records would be of limited usefulness in the standard design, at least if there were many bets, as the records would rapidly become too complex to facilitate retrieval. In short, a pair-wise design arguably offers a more propitious setting to test our theory and the effect of our interventions.

This reason also explains a second deviation from the standard methodology in the literature: in our study, participants saw relatively few pairs of bets–three, as opposed to, for example, six in [24], up to 20 in [21]. Completing pairwise combinations of tasks seems to take a greater effort than completing blocks of the same task, and to avoid fatigue and its ensuing effects on attention and hence memory, we thought that reducing the number of pairs was appropriate. This is particularly true for the recorded (T3) condition, which required participants to draw each bet observed. Doing so for any large number of tasks seemed likely to be overly tedious, and to reduce concentration on any task. On the other hand, while the low within-subject variation of stimuli makes our tests less powerful (but see below), the bet pairs we chose come from a validated domain for the effect we investigate ([23,24] both finding robust reversals in similar bets). Additionally, there seems little reason why the order and timing effects we are interested in would vary substantially across bets. Most of the variation in stimuli is likely “level” of reversal behavior, rather than “slope” or stimulus-treatment interaction with respect to order and timing. Therefore the additional benefit of a larger number of stimuli may be minimal with respect to the attentional cost they impose.

Two questions also arise concerning the distractor task and more generally potential confounds in testing EH1. First, the reader may wonder whether a 40-second task was long enough to push the attributes of previous bets out of subsequent consideration in any significant share of subjects. The evidence from such classic studies as [16,31] suggests it likely was. In Experiment 1 of [16], for instance, subjects started by reading out between 1 and 4 non-repeated pairs of consonants, presented sequentially at intervals of 0.78 sec. Then they read out 5 pairs of number digits, again shown one by one during a total time of 5 seconds. As soon as presentation was over, the subject attempted to write down each pair of consonants, in its actual order of presentation. Subjects completed several trials like this. With 3 (4) pairs, the subjects correctly recalled 40.9 (25.1) percent of the consonants in the sequence, compared with 93.5 (65.4) percent correct recall in a control condition in which the number digits were omitted, i.e., the 5 seconds interval was empty. In other words, a mere 5 seconds of distraction through an unrelated task were enough to induce very substantial forgetting. Further evidence comes from studies on free recall of elements in a list, which reveal a robust *recency effect*: items presented at the end of the list are better recalled than those in the middle, independently of the size of the list (first documented by [31]; see also e.g., [19,31]. Most relevantly, this recency effect has been shown highly vulnerable to the insertion of distracting tasks between study and test, such as solving a few mental arithmetic problems [19,25]. This hints that our intervention very likely succeeded in making subjects forget numerical details of previous bets, like their prizes and winning probabilities.

Second, if the retrieval of the alternative bet in an evaluation can be affected by the effort of attention that participants exert during prior tasks in the pair, an additional potential concern with the distractor is that subjects might (correctly) recognize the attempt to distract them, and this recognition would itself induce them to work harder to keep previously presented bets in mind. While we cannot wholly discount this possibility, it seems unlikely to us that it would create differences across treatments. To start, the distractor task was present in all treatments, and participants were not warned of its exact placing, which would make it difficult to prepare for it in advance. In addition, the mental effort of keeping previous bets in mind is cognitively similar to the mapping from keypad to dots in the distractor task, both involving the mental comparison of numbers seen elsewhere to images on the screen, so shifting attention from the distractor task to focus on previous bets should likely involve some cost in terms of performance on the sums. Participants have an incentive not to do this, as sums earn them money, and we can check in the data to see whether performance in the T2 distractor task was lower than in T1. (It was not. Indeed, participants performed somewhat better on the distractor task in T2, completing an average 11.8 sums, as opposed to 10.7 pooled across T1 and T3; combined Kolmogorov-Smirnov p > 0.1.) We are therefore relatively confident that the distractor task represented a real “cognitive obstacle” between the main tasks.

We finish with a remark on statistical power. Note that H1 and H2 can be tested with Chi-square tests on the proportion of reversals across treatments, while H3 amounts to a comparison of means. Our sample sizes are sufficient to obtain a power of 80% for such tests so long as the effect size of the proportion is at least a difference of about 16 percentage points, and the difference in average evaluation is greater than about 0.3. Identifying a 0.3-unit difference in evaluations seems to permit a reasonably sensitive test. Regarding the effect size for the reversal rates, if we judge based on [2–24] with similar bets that our T2 might result in up to 50% reversals, then so long as, for instance, the information in T3 brings the reversal rate to about 20%, and the Baseline is in between, we should have enough power for our tests. We note that this is in line with the effect size of treatment by [23], which was somewhat similar in spirit to ours. For completeness, we also report random-effect panel regressions using the bet pair as the time variable, although we note that given the low number of pairs to which our participants were exposed, the power of these tests relies crucially on the assumption that our timing and order treatments do not interact with the specific bets [32].

## 5. Conclusions

This paper uses experiments to explore a conjectured mechanism by which memory failures could be behind PRs. The argument is simple: (1) Having information about the alternative in mind during Evaluation should reduce reversal rates. (2) Temporal spacing of tasks hinders retrieval of the alternative bet during Evaluation, while a written record facilitates it. (3) Therefore, temporal spacing (whether through distractor tasks or order effects) should increase the PR rate, and a written record of previous choices should reduce it. Since conclusion (3) is not supported by the data, it appears that at least one of the premises is wrong. In this regard, our prior given the abundant evidence research on memory is that (2) is correct, and that our interventions often succeeded in pushing the alternative bet out of subsequent consideration when evaluating B, for the reasons explained in Section 2. We conclude that the idea that PRs occur when people make the evaluation of each bet without considering the alternative bet is not validated by our data. That is, it seems that memory failures concerning the alternative bet are not a major cause of PRs, at least in the classical studies.

These results contribute to the literature by offering a means to discriminate between an explanation based on unstable reference points and other well-known explanations of PRs, as the latter predict no effect of the distractor task, feedback and order on PRs. In this sense, therefore, our findings provide *indirect* evidence in favor of those theories as “alternative hypotheses”, although we clearly do not claim to have tested them directly. The regret theory of [33], for instance, posits that PRs are due to intransitivity inherent in preferences; consult [24,34,35] for experimental tests of intransitivity. [36], in turn, argue that the weighting of a bet attributes is closer to lexicographic in Choice compared to other tasks; specifically, probabilities are more heavily weighted in Choice, a phenomenon called the *prominence effect*. An additional and closely related mechanism is the *compatibility effect*, which states that responses overweight stimuli that are “compatible” with them; thus in pricing a bet (response), people will put too much weight on the attribute compatible with prices, i.e., the money payoff. In their original form, these models do not assume that attribute weights are affected by memory failures. A third variety of explanations, finally, includes theories invoking *imprecision and probabilistic preferences*. For instance, [37] assume that any given bet is associated with an “imprecision interval” of values: people can easily tell that values above the interval are preferred to the bet, and that the bet is preferred to values below. However, they find it hard to be sure whether values within the interval are “better” or “worse” than the bet. Once again, however, these theories have no place for memory or prior history without further hypotheses.

We conclude with two suggestions for further research. First, as mentioned in Table 5 above, reversals were not uniformly distributed across participants. Indeed, nearly 67% of subjects had no PRs at all, and the “worst” 4% of participants committed 25% of observed PRs. This may be suggestive for treatments that seek to reduce the incidence of PRs. Such interventions must necessarily bear upon a minority of participants, as the modal, and indeed median number of reversals is already zero.

Second, although our findings indicate that unstable reference points (in the specific sense used in this paper) do not underlie the classical PRs, this by no means implies that context is irrelevant for the occurrence of the PRs. For a clear example to the contrary, [38] asked subjects their willingness to pay (WTP) to save a number *n* of migrating birds dying in uncovered oil ponds every year. In one group of respondents, *n* was 2,000, in another 20,000, and in a third one 200,000. Yet the average WTP was similar ($80, $78, and $88, respectively) across the three groups. In contrast, one could conjecture that in a simultaneous evaluation of the three options by the same respondents, *n* would have affected the reported WTPs. Any large number of birds is difficult to evaluate independently, but in comparison their relation becomes trivial. This example neatly illustrates our point, but if the reader is interested in an example more relevant for the classical PR studies, consider the bets in pair 1 of our study (P: 8 Euros with a probability of 29/30; $: 24 Euros with a probability of 1/3). Our findings suggest that, when evaluating these bets, subjects extract hardly any insight by considering or comparing them together (perhaps they do not even attempt to do that), e.g., by checking whether the criteria used for each evaluation are consistent. Nonetheless, consider an alternative scenario where the P-bet is substituted by a bet similar to the $-bet, e.g., 24 Euros with a probability of 2/3. We submit that in this case subjects with a modicum of attention would try to be consistent across valuations, i.e., the certainty equivalent of the bet with higher winning probability should be higher. If such type of referents were numerous enough and subjects had them in mind, moreover, we believe that PRs would decrease.

This conjecture seems in line with the evidence from [34], where certainty equivalents are elicited either by (i) asking subjects the amount of money *x* that leaves them indifferent between *x* and the gamble, or by (ii) an iterated procedure in which subjects are first asked to give their preference between the gamble and a fixed sum of money; if the subject prefers the gamble (money), the question is made again, but the amount of money is increased (decreased) by $0.04; this series of questions is repeated until the preference changes. [34] reports that the frequency of PRs drops substantially when procedure (ii) is used. It is also in line with the admittedly weak evidence we found in the disaggregate analysis on the differences between the “easier” and “more difficult” bet pairs, e.g., Pair 3 and Pair 1 in our experiment, respectively, see the discussion at the end of Section 3. We hence conclude with a conjecture for further research: context matters for PRs, but mostly when subjects have it in mind and it is simple enough, i.e., when the insights derived from the comparisons are easy enough to mentally reconstruct.

## Supporting information

S1 Fig Appendix Fig I. Original evaluation screenshot (for the French sessions).
(TIF)

S2 Fig Appendix Fig II: Original choice screenshot (for the French sessions). (TIF)

S1 File Raw data. (DTA)

S2 File Analysis program (original submission). (DO)

S3 File Online Appendix: Instructions, screenshots and alternate history regressions. (DOCX)

S4 File Regressions excel sheet. (XLSX)
