# Supplementary figures and images for: Easier comparison of bets in evaluation does not reduce classical preference reversals: Evidence against a context-dependent explanation
Source: PLoS One. 2024 Jan 3;19(1):e0292011. doi: 10.1371/journal.pone.0292011 (PMC10763930; doi:10.1371/journal.pone.0292011)

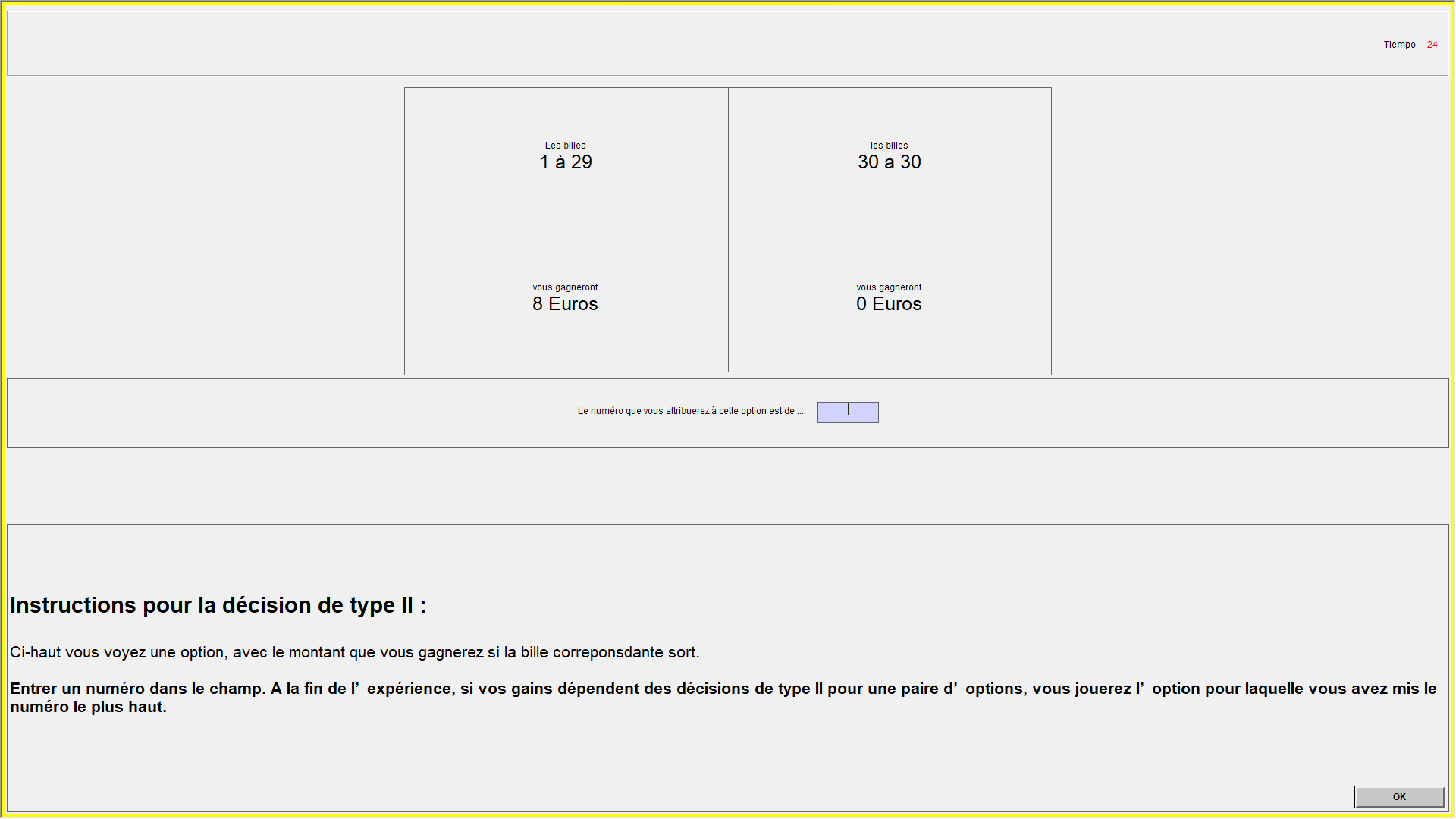

Supplement: S1 Fig — Original evaluation screenshot (for the French sessions). (TIF) [file pone.0292011.s001.tif]

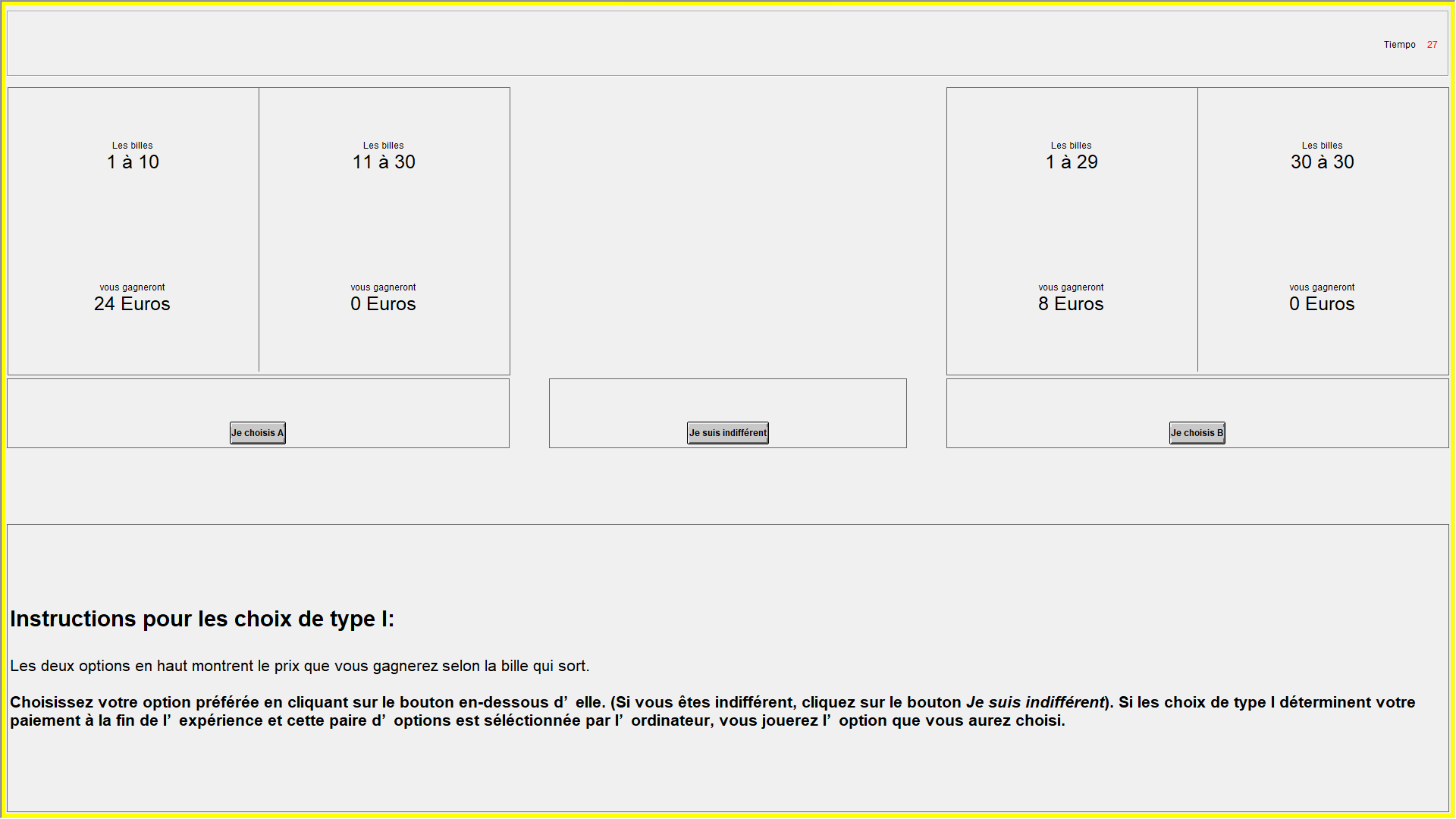

Supplement: S2 Fig — (TIF) [file pone.0292011.s002.tif]
