# Supplementary material for: Easier comparison of bets in evaluation does not reduce classical preference reversals: Evidence against a context-dependent explanation
Source: PLoS One. 2024 Jan 3;19(1):e0292011. doi: 10.1371/journal.pone.0292011 (PMC10763930; doi:10.1371/journal.pone.0292011)
Supplement: S3 File — (DOCX) [file pone.0292011.s005.docx]

**Online appendix to “Easier comparison of bets in evaluation does not reduce classical preference reversals: Evidence against a context-dependent explanation”**

# Translated instructions

Thank you for participating in this experiment on choice under uncertainty. At the end of the experiment, you will be paid some money; the precise amount will depend on chance and your decisions. In this respect, we note that there are no trick questions, and no wrong answers: Just choose what you prefer. Your decisions will be anonymous, that is, participants will get no information on other participants’ decisions.

You will take decisions with your computer keyboard and mouse. Read carefully at any moment all the information appearing in your screen. Except in a few screens, there is no hurry to decide. In fact, to move on from some screen to the next it is necessary that all other participants have made their choice in the first screen.

It is very important that you do not talk to any other participant. If you do not follow this rule we will have to exclude you from the experiment. If you have questions, please raise your hand and we will assist you.

Description of the Experiment

In this experiment we speak of **options** or alternatives. When choosing an option there are two possible **outcomes**: (i) Earn some money, (ii) earn nothing. Each outcome has a **probability**, described with reference to a random draw of a ball from a bag with 30 numbered balls. Example of an option (see figure below): You win 8 Euros if balls 1 to 20 are drawn and 0 Euros otherwise. The probability of winning in this option is 20/30.

**Example of option**

:


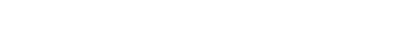


Ball 21 to

30


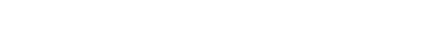


Outcome: You earn

Euros

8


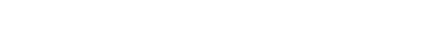


Ball 1 to

20


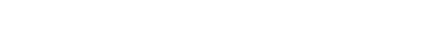


Outcome: You earn nothing

On the other hand, during the experiment there are two types of decisions:

- **Type I**: The screen shows a pair of options and you must choose your favorite. If you are indifferent between them, you can also express it.
- **Type II**: The screen shows one option, and you must state the maximum amount of money that you would bet to play it ‒in other words, the maximum price that you would pay to play it.

There are a total of six different options, grouped in three different pairs. For each pair of options (A and B) you will take three decisions, consecutively but in random order: (i) Decision type I between A and B, (ii) decision type II for A, (iii) decision type II for B. For any decision, you will be able to observe its type in the screen; a short reminder describing the decision will be also provided. [**Treatment 3**: Before you introduce your choice in the screen, you are asked in each decision to draw the option or options available. The drawing for each option must follow the scheme of the prior example. Use the blank sheets over your table for the drawing. For type I decisions, indicate your favorite option as well. For Type II decisions, also indicate the maximum price that you would pay to play it.]

| **Options** | A1 | B_1_ | A2 | B_2_ | A3 | B_3_ |
| --- | --- | --- | --- | --- | --- | --- |
| **Pairs** | P1 | | P_2_ | | P_3_ | |
| **Decisions** | 1. decision type I 2. decisions type II | | 1. decision type I 2. decisions type II | | 1. decision type I 2. decisions type II | |

Apart from decisions I and II, you will also have to answer a sequence of calculation exercises. For these, you will be shown a 3x3 grid with one or more dots in some cells (see the left-hand figure). Assuming that the grid represents the calculator keypad on your keyboard (see the right-hand figure), you will have to assign to each dot its cell number, mentally add all those numbers, and enter the sum in a box in the screen. If this sum is correct, you will move on to the next exercise. In the example below, the sum would be 1 + 5 + 6 + 6 = 18. During the experiment, you will have 2 minutes in total to complete as many of these exercises as you can, and will be paid 50 cents for each pair of sums correctly entered. For instance, if you complete seven sums correctly, you will earn € 1.50 from this part of the experiment.

|  |  |  |  | 7 | 8 | 9 |
| --- | --- | --- | --- | --- | --- | --- |
|  | 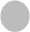 | 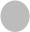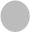 |  | 4 | 5 | 6 |
| 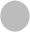 |  |  |  | 1 | 2 | 3 |

**Grid with dots** **Calculator keypad**

Once you have made the nine type I and II decisions (three for each pair of options), the computer will select randomly one of the three pairs (call it P) and one decision type (I or II). All this will then determine one of the six options, the only one with which you can earn money. More precisely:

- If the computer chooses type **I**, the selected option will be the one that you chose as favorite in your decision type I for P ‒ if you expressed indifference in that decision, you will play the option in P randomly selected by the computer. Your payoff will be determined by the computer randomly generating a number from 1 to 30; you will get the corresponding prize if the ball drawn is a ‘winning’ ball.

- If the computer selects type **II**, the selected option will be the one for which you bet more in the two decisions type II in P. If both bets were equal, the computer will randomly select one of the options in P.

After completing a short, anonymous questionnaire, you will be paid privately in cash in an adjoining office. Your final payment will be computed as follows.

Your payoff from a single option (selected as explained before) + payoff from the calculation exercises + 4 Euros for participating in the experiment.

**Before we start the experiment, please answer the following questions. Raise your hand when you are done so that we can verify the answers.**

- How many different options are there in this experiment?______________

- A number of these options will be randomly chosen at the end of the experiment to play for real money. How many options will you end up playing for real money? _____________

In a hypothetical example, consider an option A that gives ________ Euros (choose a number to construct your own example) if a ball from 1 to ____ (choose a number from 1 to 30) is selected, and zero otherwise. Suppose that this option A is finally selected for play. For this hypothetical example,

- What would be your probability of winning for this option? _______

Suppose that option A in the previous example is paired with another option B that gives _________ Euros if a ball from 1 to _____ is selected (choose numbers so as to construct your own example), and zero otherwise. In the type I decision, you choose option A B (select your preferred option), and in the type II decisions, you are willing to pay a maximum price of ______ Euros for option A and ______ Euros for option B (indicate each price). Assume that this pair A-B is randomly chosen at the end of the experiment.

- If in addition the type I decision is randomly chosen, what option will you play? __________
- If on the contrary the type II decision is randomly chosen, what option will you play? __________

[**Treatment 3**: Draw options A and B from your example according to the scheme used in the instructions above.]

# Interface screen shots


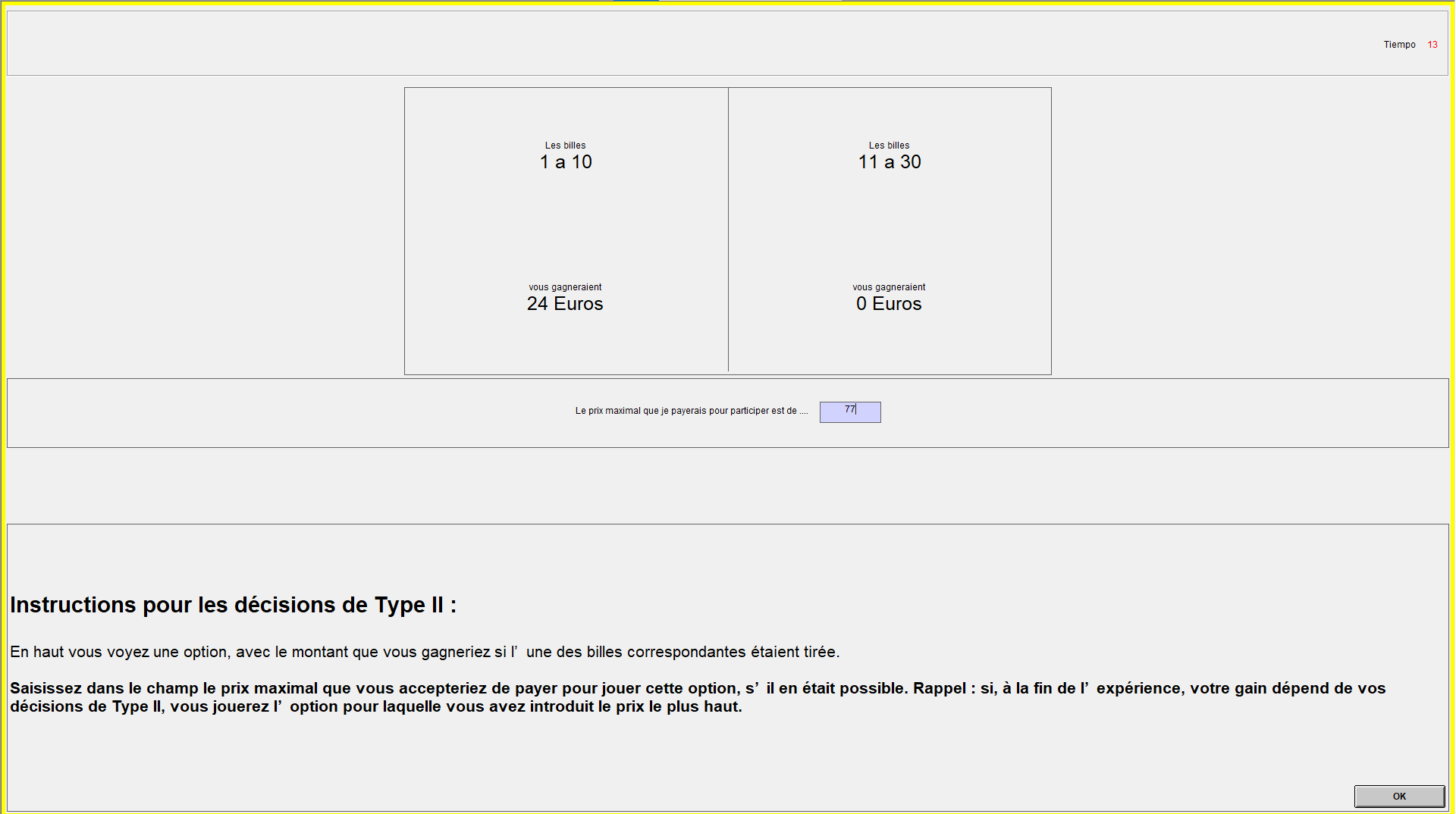


**Fig I**: Original evaluation screenshot (for the French sessions)


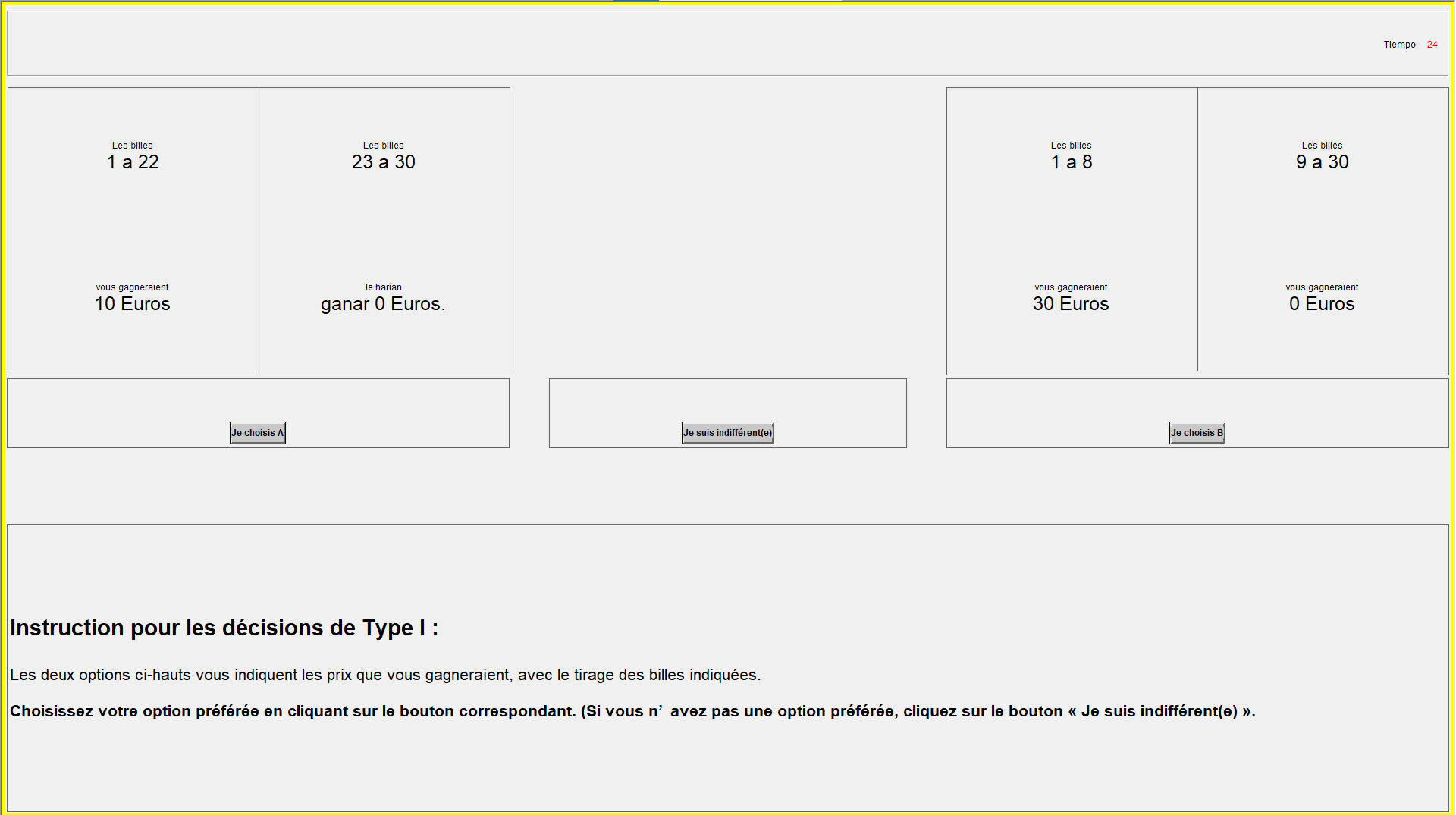


**Fig II**: Original choice screenshot (for the French sessions)

# Alternative analysis of order effects on evaluations and likelihood of PRs

Our definition of a history is naturally one of many possibly alternatives. For robustness, we explore a variation below that highlights a recency effect, i.e., whether order effects on PRs and evaluations depend only on the task that immediately precedes the given evaluation. So instead of analyzing the effect of “both other tasks” (see Table 7 in the paper), we study just the effect of the previous task. Table I compares the new set of considered histories (see the two columns on the right), with the histories considered in the paper (columns 3 and 4). We are thankful to one referee for this suggestion.

| Order | Sequence of tasks | (3)  $-bet history in paper | (4)  P-bet history in paper | (5) Alternative $-bet history | (6) Alternative P-bet history |
| --- | --- | --- | --- | --- | --- |
| 1 | C-$-P | Choice | Both | Choice | $-bet |
| 2 | C-P-$ | Both | Choice | P-bet | Choice |
| 3 | $-P-C | Nothing | $-bet | Nothing | $-bet |
| 4 | $-C-P | Nothing | Both | Nothing | Choice |
| 5 | P-$-C | P-bet | Nothing | P-bet | Nothing |
| 6 | P-C-$ | Both | Nothing | Choice | Nothing |

**Table I**: alternate history definition

The regression analysis is reported in Table II, which is somehow analogue to Table 8 in the paper. Regressions (1) to (3) explore the effect of task prior to Evaluation of the P-bet (if any), while regressions (4) to (6) consider the task coming immediately before Evaluation of the S-bet (if any).

The analysis shows that the value of the $-bet (but not the P-bet) is significantly raised by completion just after Choice, as both alternative coefficients are significantly negative and not significantly different from each other. However, this does not affect the likelihood of either standard or non-standard reversals.

| **VARIABLES** | **(1)** | **(2)** | **(3)** | **(4)** | **(5)** | **(6)** |
| --- | --- | --- | --- | --- | --- | --- |
|  | **Evaluation of P-bet** | **Strict standard PR** | **Strict non-standard PR** | **Evaluation of $-bet** | **Strict standard PR** | **Strict non- standard PR** |
| **P preceded by $** | 0.570 | 0.00367 | -0.0901 |  |  |  |
|  | (0.981) | (0.306) | (0.360) |  |  |  |
| **P first** | -0.678 | 0.136 | -0.437 |  |  |  |
|  | (0.838) | (0.279) | (0.351) |  |  |  |
| **$ preceded by P** |  |  |  | -1.929* | -0.459 | -0.0941 |
|  |  |  |  | (0.884) | (0.288) | (0.375) |
| **$ first** |  |  |  | -2.428** | -0.163 | -0.501 |
|  |  |  |  | (0.840) | (0.287) | (0.363) |
| **Observations** | 870 | 870 | 870 | 870 | 870 | 870 |
| **Number of PID** | 290 | 290 | 290 | 290 | 290 | 290 |
| **Note:** Coefficient estimates for random effects OLS (models 1 and 4) and logistic regressions (remaining models). Robust standard errors in parentheses. Controls include scores on the CRT and distractor task, as well as age, gender, a dummy for the French sessions, and indicators for the different bets. ** p < 0.01, * p < 0.05. | | | | | | |

**Table II** regression results on order effects.
